# Visual assessment of antimicrobial medicine packaging and labeling quality in pharmacies of Ho Municipality, Ghana

**DOI:** 10.1371/journal.pone.0342484

**Published:** 2026-02-13

**Authors:** Emmanuel Orman, Bridget Dzidzinu Ankah, David Oteng, David Mccarthur, Thelma Alalbila Aku, Araba Ata Hutton-Nyameaye, Jonathan Jato, Hayford Odoi, Samuel Owusu Somuah, Issaka Nii Amu Collison-Cofie, Yogini H. Jani, Cornelius Dodoo

**Affiliations:** 1 School of Pharmacy, University of Health and Allied Sciences, Ho, Ghana; 2 Pharmacy Department, Ho Teaching Hospital, Ho, Ghana; 3 Food and Drugs Authority, Ho, Ghana; 4 School of Pharmacy, University of London, London, United Kingdom; 5 Centre for Medicines Optimisation Research and Education, UCLH NHS Foundation Trust, London, United Kingdom; Debre Berhan University, Asrat Woldeyes Health Science Campus, ETHIOPIA

## Abstract

**Background:**

Substandard and falsified antimicrobials threaten public health due to their role in resistance development. Pharmacies, as key access points for antimicrobials, can play a crucial role in detecting these products. In this study, a visual assessment tool which incorporates a novel packaging quality index estimate, was developed and used in pharmacies to evaluate antimicrobial packaging and flag suspicious products.

**Methods:**

A cross-sectional study was conducted in 23 community pharmacies in the Ho Municipality between November 2023 and February 2024. The developed checklist contains indicators on registration compliance, language & medical information, batch information consistency, and product security of the antimicrobials. The tool was validated, and its use on randomly sampled antimicrobials informed the development of the packaging quality index from regression analysis involving weights determined from principal component analysis of the results. Statistical analyses were performed with SPSS (version 26.0) and OriginPro (version 2022).

**Results:**

The packages and labels of the 275 antimicrobials evaluated were antibacterials (41%), antiprotozoals (28%), antifungals (22%) and antivirals (9%). Most products were of foreign origin (58.5%) and labelled in English (98%). Significant variations were observed in the registration compliance and product security indicators by origin and antimicrobial class (*p* < 0.01). Batch information consistency varied significantly across the antimicrobial classes (*p* < 0.01), whereas language & medical information quality remained consistent. The packaging quality index scores followed a normal distribution, with majority (95.6%) referred to as moderate quality (Index: 1.81–5.34). High quality packages (2.2%; Index: 5.47–5.53) were mostly observed in antibacterials of local origin (66.7%) whereas the poor-quality packages observed (2.2%; Index: 1.48–1.78) were mostly antibacterials of foreign origin (66.7%).

**Conclusion:**

This study identified packaging quality issues among the antimicrobials investigated which may suggest potential risk to falsification. Routine antimicrobials packages assessment with developed tools like the packaging quality index could help pick up early signals of substandard and falsified antimicrobials for further regulatory investigations and action.

## 1. Introduction

The availability and accessibility of safe and effective antimicrobials play a crucial role in combatting infectious diseases and protecting public health worldwide [1]. This objective is, however, threatened by the presence of substandard and falsified (SF) medicines. SF medicines may be disguised versions of established brands, or authentic products that do not meet the required quality standards or specifications [2]. They may contain incorrect ingredients or quantities thereof, or may have no active ingredients at all [3,4]. They are very often improperly formulated or manufactured under substandard conditions [5]. As a result, SF products tend to misrepresent their identity, quality, and efficacy to consumers [5,6].

According to the World Health Organization (WHO), 10% to 15% of all pharmaceuticals worldwide are counterfeit [7,8]. However, the prevalence of counterfeits is much higher in countries with limited regulatory capacity, accounting for up to 50% of available medicines, while developed regions like North America and Western Europe have a lower percentage [9]. Africa contributes to about 42% of global cases of counterfeit drugs, with an estimated market value of around $200 billion [10]. The problem in Africa is worsened by challenges such as poor drug traceability, inadequate infrastructure, and ineffective border control, compounded by a lack of comprehensive data to fully understand the extent of the issue [11].

The presence of SF medicines poses significant risks to patient safety and exacerbates the challenges faced by national healthcare and medicine regulatory systems [12]. Effects such as increased out-of-pocket expenditure on healthcare treatment, loss of confidence in the healthcare system, and most importantly, treatment failure, which includes antimicrobial resistance, and toxic side effects when harmful substances are present in these products, have been documented [10,13].

Traditionally, SF medicines may be detected using physical and chemical analytical methods, which authenticate the chemical composition, assay their content and assess their purity status [14–17]. Access to these techniques is limited by high cost of purchase, installation, and maintenance, and the need for trained personnel to conduct the analyses, especially in resource-limited settings, like Ghana, where the problem of SF medicines is often most pronounced [18].

The lack of sufficient analytical resources to assess suspected SF medicines highlights the necessity to reevaluate the quality assurance strategies implemented to protect public health and ensure the safety of medicine consumption [19,20]. According to Dégardin & Roggo [21], an evaluation of the packaging of 20 seized medicines on suspicion of being fake turned out to be beneficial. All except one of the packages were discovered to be fake after the study.

Though valuable, visual assessments of the packages and labels medicines often rely on subjective judgement, which can lead to inconsistent results and reduce the chances of detecting preliminary signals of potential SF medicines. Additionally, the reproducibility of the assessment across different assessors and settings is limited [22,23]. A tool with a quantifiable evaluation framework to enhance early detection of potentially SF medicines using product packaging quality would complement the existing visual examination methods with objectivity, and enhanced reliability. It will also provide a cost-effective alternate means of identifying pharmaceuticals that require further analysis, especially in resource-challenged settings where sophisticated analytical resources may not be easily accessible.

Therefore, the aim of this study was to develop and use a visual, objective and reproducible assessment tool to evaluate pharmaceutical packaging quality and monitor quality-indicating parameters of product packages and labels. The tool is proposed to be cheap, easy to use and beneficial in serving as early detection signal to call for thorough analytical investigations of suspected medicines.

## 2. Materials and methods

### 2.1. Study design and setting

#### 2.1.1. Type of study and duration.

This was a cross-sectional observational study designed to use a visual assessment checklist that was developed to assess key quality attributes and information on packages and labels of antimicrobial medicines in the community pharmacies. The study took place between November 2023 and February 2024.

#### 2.1.2. Study sites.

The study was conducted in the Ho Municipality, located in the Volta Region of Ghana. Ho serves as the capital of both the municipality and the region, making it a critical location for healthcare service delivery. According to the records of the Volta Regional Branch of the Pharmacy Council of Ghana, Ho has the highest concentration of community pharmacies in the region. These pharmacies serve as key sources of medicines for the population, and thus, are expected to play a significant role in ensuring the authenticity and safety of antimicrobial drugs.

#### 2.1.3. Inclusion and exclusion criteria.

All community pharmacies officially registered by the Pharmacy Council of Ghana by June 2023 and operational in the Ho municipality and gave their written consent through their proprietors, proprietresses or superintendent pharmacists, were included in the study. Hospitals, licensed chemical shops, and herbal shops were excluded. The study focused exclusively on antimicrobial drugs listed in the Essential Medicines List of Ghana (MOH, 2017), including antivirals, antibacterials, antifungals, and antiprotozoans. Herbal or cosmeceutical product and galenicals with antimicrobial effects were excluded.

### 2.2. Sampling

At the time of the study, there were thirty-five (35) registered and operational community pharmacies in the municipality. Hence, a total population sampling was considered. However, out of the 35 pharmacies, 23 gave their written consent for participation in the study. To ensure adequate representation of each of the four main classes of the antimicrobials, a stratified simple random sampling approach was adopted. An average of 12 antimicrobial drugs were examined in each facility, including three each of antivirals, antibacterials, antiprotozoals, and antifungals. The drugs were selected randomly from the available stock at each facility. Samples of the same brand or from the same manufacturer were only included if their batch numbers differed. If a randomly chosen sample was found to have the same batch number as a previously assessed one, whether from the same or a different pharmacy, that sample was excluded, and a new random selection was made.

### 2.3. Data collection

The visual assessment tool designed for this study was adapted from a previous tool developed by Schiavetti *et al*. (2020) with additional indicators informed by the labeling requirements of the Food and Drugs Authority (FDA), Ghana. The checklist was written in English and contained four main sections: 1) general information about the medicine being examined such as name of product, dosage type, manufacturer, registration status in Ghana; 2) quality of medical information on the package; 3) batch information consistency; and 4) security features and their traceability [S1 Table**]**. The study team and 3 regulatory experts reviewed the content for validity and relevance. Face validity was also confirmed during a stakeholder meeting with pharmacy staff, where the tool was tested on ten different antimicrobials. To test for reliability, two assessors from the team, in a pilot study, used the revised tool to independently evaluate five similar antimicrobials in one of the pharmacies. The inter-assessor agreement was shown to very high (% = 99.23%; Cohen’s κ = 0.9751), proving the reliability of the assessment tool [24].

### 2.4. Development of the Packaging Quality Index (PQI)

The Packaging Quality Index (PQI) was developed as a composite quantitative measure to objectively assess the quality of antimicrobial medicine packages and labels based on key regulatory and quality-indicating packaging indicators. Parameters included in the visual assessment tool [S1 Table] were scored using predefined binary or ordinal scales [S2 Table]. Higher weights were assigned to more critical regulatory components identified through principal component analysis (PCA) of cumulative scores across various categories, including registration compliance scores, language and medical information quality scores, batch information consistency scores, and product security scores [S3**-**S5 Tables]. The regression model developed for the estimation of the PQIs of the products has the general formula shown in Equation 1. PQI scores were shown to follow a normal distribution, and a three-class quality classification system, (high quality, moderate quality, and poor quality), was established for the packaging quality of the products based on PQI scores. Internal consistency checks validated the PQI model, by confirming that products classified as high quality consistently exhibited superior packaging attributes, to support its reliability for regulatory and quality control purposes.


$$\begin{array}{cc} & Packaging\;Quality\;Index\;(PQI)=\;w_{\text{1}}(Registration\;Compliance\;Score)\\ & +w_{\text{2}}(Language\;\&\;Medical\;Information\;Quality\;Score)+w_{\text{3}}(Batch\;Information\;Consistency\;Score)\\ & +\;w_{\text{4}}(Product\;Security\;Score) \end{array}$$ (1)

where w_1_, w_2_, w_3_, and w_4_ represent the PCA-based weights or coefficients for each component score.

### 2.5. Data handling and analysis

Data was recorded electronically using an assessment form designed with EpiInfo software (version 7.2.6.0, CDC, 2023). Upon completion, the electronic records were exported into Microsoft Excel (Microsoft 365, Version 2410) and reviewed for completeness and accuracy before the data were analysed. The data was then exported into SPSS (version 26.0, 2019) and OriginPro (OriginLab Corporation, version 2022) for further analysis. To ensure consistency, the data were cross-checked against the original checklists. Any discrepancies or missing data were once more addressed. All identifying information linked to specific pharmacies were anonymized prior to analysis. Descriptive and inferential statistics were used to analyze the findings. Regression analyses were conducted to assess the relationship between the factors under the background of the products and both categorical and numerical data from the different categories of assessment parameters. Principal component analysis was used to explore the variability in the scores data leading to the determination of the coefficients used to develop the PQI. Statistical significance was determined at a *p*-value of less than 0.05.

### 2.6. Ethical considerations

Ethical approval for this study (UHAS-REC A.9 [68] 22–23) was granted by the University of Health and Allied Sciences Research Ethics Committee. Only pharmacies that provided informed written consent through their proprietors, proprietresses or superintendent pharmacists were included in the study.

## 3. Results

### 3.1. Background information on products

**Fig 1:**
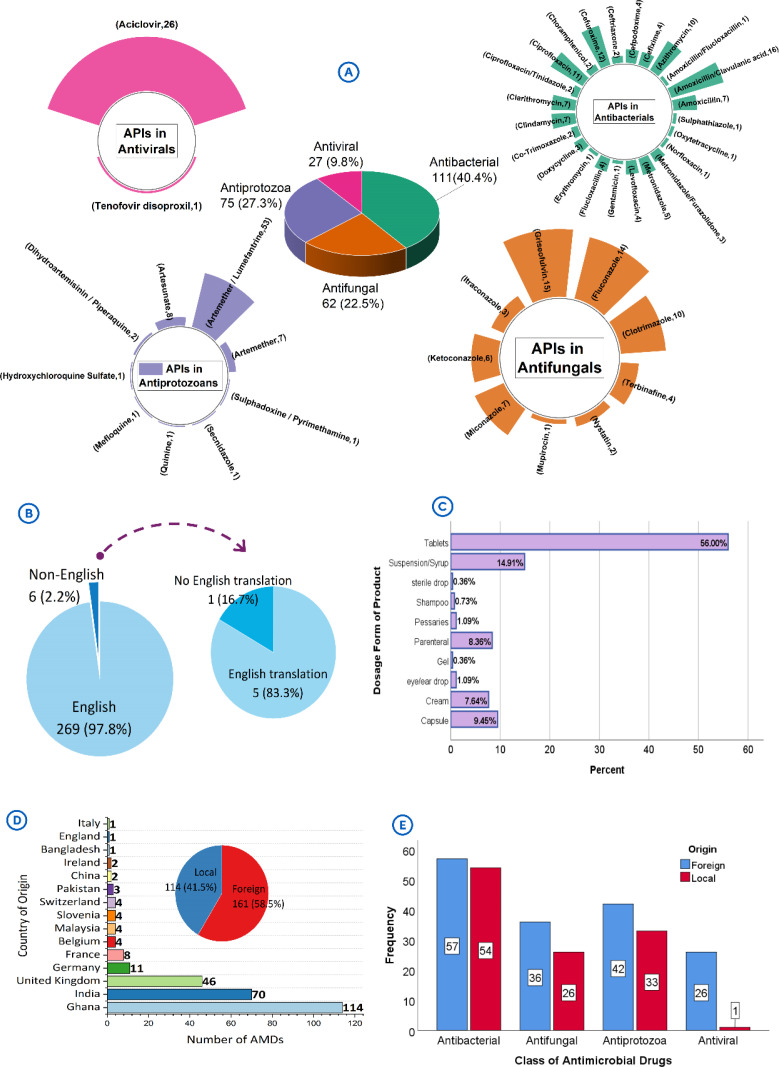
Background description of antimicrobial samples investigated in the study. **[A]**: Breakdown on the different antimicrobial classes and their active pharmaceutical ingredients. **[B]**: Language used in writing information on the packages and labels. **[C]**: Dosage forms of the samples. **[D]**: Origin of the samples. **[E]**: Comparison of the class of the antimicrobials by their origin. *Legends*: API – Active pharmaceutical ingredient; AMDs – antimicrobial drugs.

The packaging and labelling contents of 275 antimicrobial medicines comprising, antibacterials (n = 111/275, 40.4%), antifungals (n = 62/275, 22.5%), antiprotozoals (n = 75/275, 27.5%) and antivirals (n = 27/275, 9.8%) were assessed (Fig 1a). Most of the products contained single active pharmaceutical ingredients (APIs) (n = 194/275, 70.5%). For the antibacterials, the prevalent API compositions were amoxicillin/clavulanic acid (n = 16/111, 14.4%), cefuroxime (n = 12/111, 10.8%), ciprofloxacin (n = 11/111, 9.9%) and azithromycin (n = 10/111, 9.0%). Griseofulvin (n = 15/62, 24.2%), fluconazole (n = 14/62, 22.6%) and clotrimazole (n = 10/62, 16.1%) constituted the most APIs in the antifungals. In the case of the antiprotozoals, the products contained mainly artemether/lumefantrine (n = 53/75, 70.7%), artesunate (n = 8/75, 10.7%) and artemether (n = 7/75, 9.3%), whereas for the antivirals, only two types of APIs were found, and they were aciclovir (n = 26/27, 96.3%) and tenofovir disoproxil (n = 1/27, 3.7%).

Majority of the products had packaging information written in English (n = 269/275, 97.8%) (Fig 1b). Most of the products assessed presented either as tablets (n = 154/275, 56.0%), oral liquids (syrups & suspensions) (n = 41/275, 14.9%), capsules (n = 26/275, 9.5%), parenterals (n = 23/275, 8.4%) or topical creams & ointments (n = 21/275, 7.6%) (Fig 1c). Most of the products were of foreign origin (n = 161/275, 58.5%; χ2 = 8.0327, p = 0.005) and predominantly imported from India (n = 70/161, 43.5%), and United Kingdom (n = 46/161, 28.6%) (Fig 1d). The origins of the different antimicrobial classes were found to be significantly different (χ2 = 18.43, p = 0.0004), and this was mostly due to the disproportionately high proportion of foreign antivirals assessed (n = 26/27, 96.3%) (Fig 1e).

### 3.2. Registration compliance of the products

**Fig 2:**
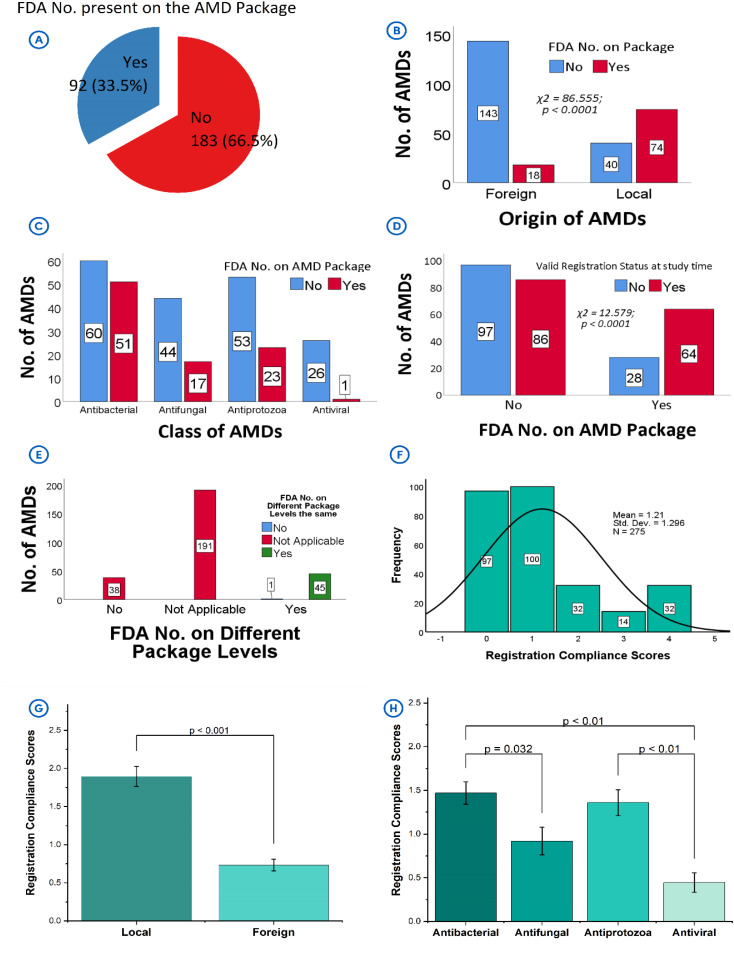
Registration status of samples investigated. **[A]**: Presence of FDA registration number on the package of the product. **[B]**: Presence of the FDA number on product package with respect to product origin. **[C]**: Presence of the FDA number on product package with respect to antimicrobial class. **[D]**: Relationship between the presence of the FDA number and whether the product held a valid registration status at the time of the study. **[E]**: Presence of the FDA number on different packaging levels of the product and whether the numbers present are the same at all levels. **[F]**: Distribution of registration compliance scores attained by products. **[G]**: Comparing the registration compliance scores of products by their origin. **[H]**: Comparing the registration compliance scores by their antimicrobial class. *Legends*: API – Active pharmaceutical ingredient; AMD(s) – antimicrobial drug(s); FDA – Food and Drugs Authority of Ghana.

A significantly higher proportion of the products (n = 182/275, 66.5%, χ^2^ = 30.11, *p* < 0.0001) (**Fig 2a**) had no FDA registration numbers displayed on their packages, with the compliance level seeming better among products from local origin (n = 74/114, 64.9%) as compared to those of foreign origin (n = 18/161, 11.2%; χ^2^ = 86.56, *p* < 0.0001) (**Fig 2b**). Significant variation in the presence of the registration numbers across different antimicrobial classes (χ^2^ = 18.23, *p* < 0.0001) was also observed (**Fig 2c**). Although there were relatively higher proportions of non-compliant products in relation to the registration number in each class, the values were more prominent among the antivirals (96.3%), antifungals (71.0%) and antiprotozoals (69.3%). A notable proportion of the products were without valid registration status (n = 125/275, 45.5%) at the time of the study (**Fig 2d**). Further analysis identified the presence of the registration number as a strong predictor of the validity of the product’s registration status [χ^2^ = 12.58, OR=2.58 (1.52–4.38), *p* < 0.0001], reinforcing the importance of displaying registration details on packaging. A small proportion of the products displayed their registration numbers (n = 46/275, 16.7%) across the three different package levels (**Fig 2e**), which were found to be very consistent (Pearson’s *r* = 0.7409, χ^2^ = 267.98, *p* < 0.0001).

The above findings strongly aligned with the Registration Compliance Scores, which provided quantitative estimates to the categorical variables. While the regulatory compliance scores were low [mean = 1.21 ± 1.30; maximum expected score (MES)=4] (**Fig 2f**), products with displayed FDA registration numbers, consistent across multiple packaging levels and with valid registration status at the time of the study, achieved higher scores. This indicated their superior compliance with regulatory standards. In contrast, the absence of FDA numbers or discrepancies across levels contributed to relatively lower scores. This was evident in the significantly higher average scores for the locally manufactured products (1.89 ± 1.40) compared to the lower scores for those of foreign origin (0.73 ± 0.96; *p* < 0.0001) (**Fig 2g**). Similarly, the scores for the different antimicrobial classes were significantly different from each, with that of antibacterials being the highest (1.47 ± 1.36), followed by antiprotozoals (1.36 ± 1.29) and antifungals (0.92 ± 1.25). The antivirals recorded the least score (0.44 ± 0.58, *p* < 0.0001) (**Fig 2h**).

### 3.3. Language and information quality

**Fig 3:**
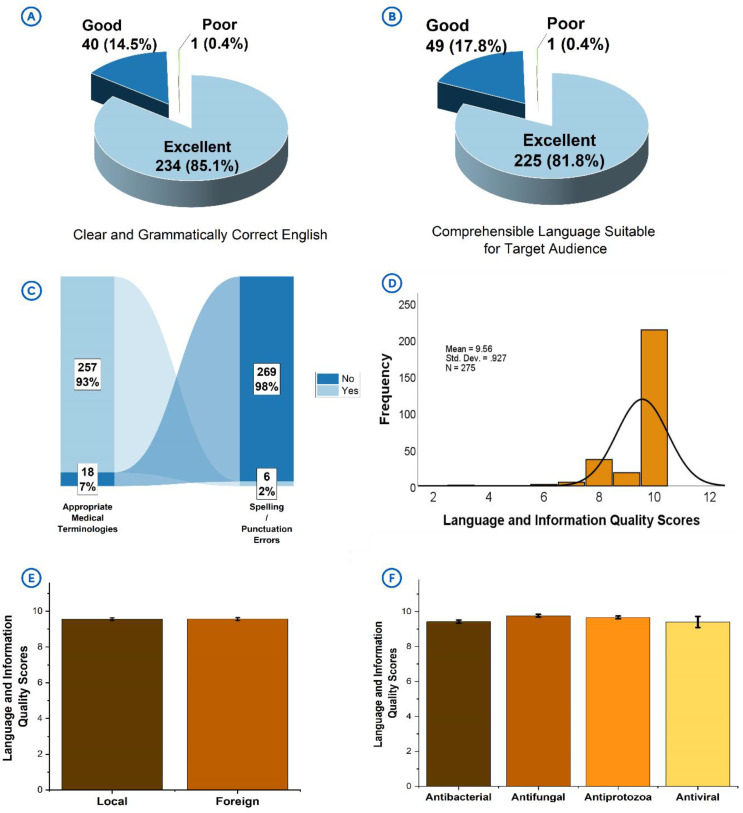
Quality of language and medical information on product packages. **[A]**: Presence of clear and grammatically correct English language. **[B]**: Use of language, comprehensible and suitable for the target audience. **[C]**: Appropriateness of terminologies, and presence of spelling or punctuation errors. **[D]**: Distribution of language and information scores attained by products. **[G]**: Comparing the language and information scores of products by their origin. **[H]**: Comparing the language and information scores by antimicrobial class.

The language used on products was observed to be generally clear and grammatically correct (n = 274/275, 99.6%) (**Fig 3a**) as well as comprehensible and suitable for the end users (n = 274/275, 99.6%) (**Fig 3b**). Most of the medical terminologies used were appropriate and without ambiguity (n = 257/275, 93.5%) (**Fig 3c**) and without spelling and punctuation errors (n = 269/275, 97.8%) (**Fig 3d**). These observations translated in the products recording consistently high language and information quality scores [mean = 9.56 ± 0.93; MES = 10] (**Fig 3d**). Product origin (**Fig 3e**) and antimicrobial class (**Fig 3f**) had no significant effect on the scores attained.

### 3.4. Batch information consistency

**Fig 4:**
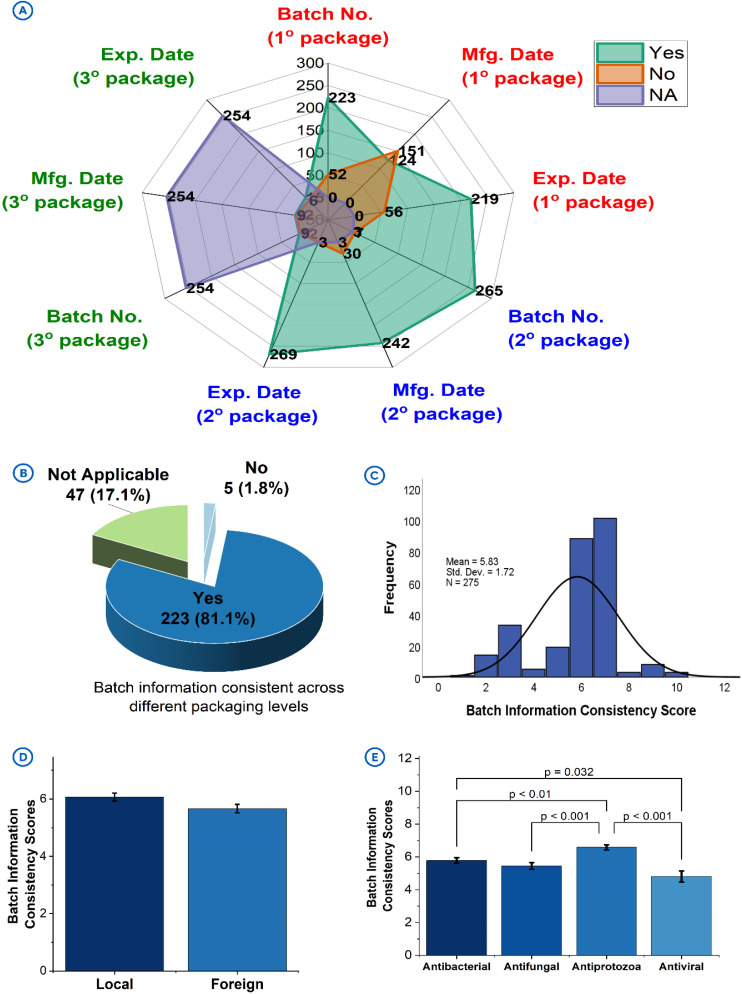
Batch information consistency on product packages. **[A]**: Presence of batch information on primary, secondary, and tertiary packages of products. **[B]**: Consistency of batch information across different packaging levels of products. **[C]**: Distribution of batch information consistency scores attained by products. **[D]**: batch information consistency scores by product origin. **[E]**: batch information consistency scores by antimicrobial class.

Batch information include batch numbers, manufacturing dates and expiry dates. These are critical components of product packaging [25]. The current study observed that batch information were present on the primary, secondary, and tertiary product packages. While their presence on the primary package was deemed satisfactory (batch no.: n = 223/275, 81.1%; man. date: n = 124/275, 79.6%; exp. date: n = 219/275, 79.6%), their presence on the secondary packages was rated better (batch no.: n = 265/275, 96.4%; man. date: n = 242/275, 88.0%; exp. date: n = 269/275, 97.8%) and poorer on the tertiary packages (batch no.: n = 12/275, 4.4%; man. date: n = 12/275, 4.4%; exp. date: n = 15/275, 5.4%) (**Fig 4a**).

The consistency of batch information across different packaging was noteworthy (n = 223/275, 81.1%) (**Fig 4b**), although the mean batch consistency score was 5.83 ± 1.72, (with an MES of 10) (**Fig 4c**), indicating an average performance. This could be due to the unavailability of some packaging levels for assessment (n = 44). Even though the local products recorded batch information consistency scores (mean = 6.07 ± 1.47) comparable to that of the foreign products (mean = 5.66 ± 1.86, *p* = 0.0539) (**Fig 4d**), all the products with inconsistent information were also found to be of local origin (n = 5/5, 100%). Antiprotozoals recorded the highest batch information consistency scores (mean = 6.59 ± 1.37), followed by antibacterials (mean = 5.78 ± 1.79), antifungals (mean = 5.45 ± 1.62) and then antivirals (mean = 4.81 ± 1.75) (**Fig 4e**).

### 3.5. Product security

**Fig 5:**
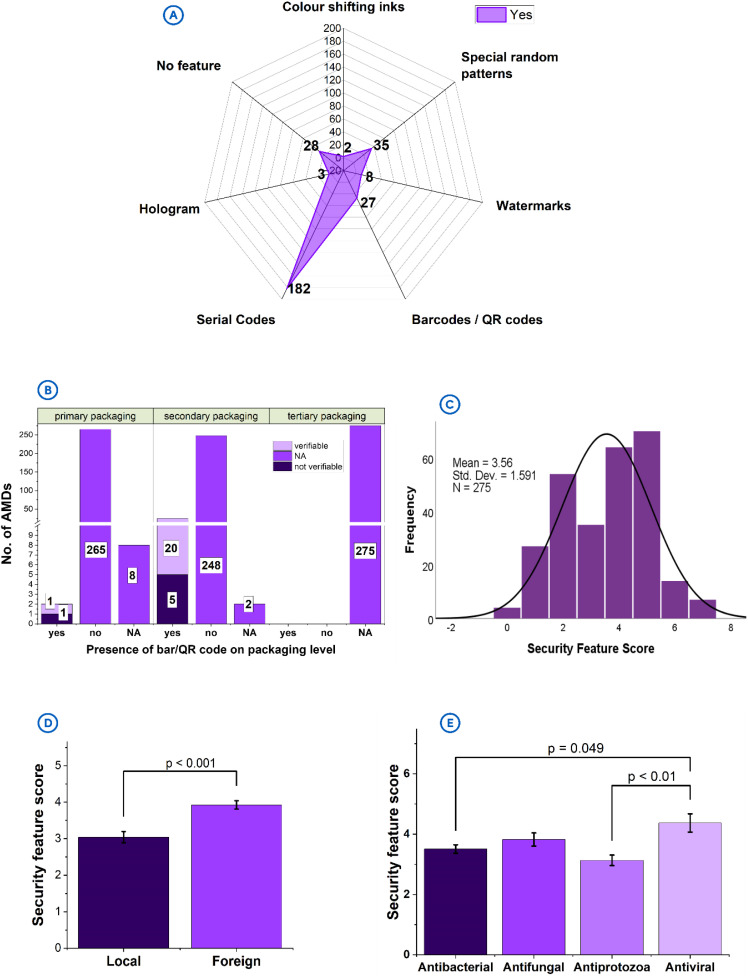
Product security features on packages. **[A]**: The different security features incorporated into products packages. **[B]**: Presence of bar/QR codes on different packaging levels and their verifiability through scanning. **[C]**: Distribution of product security scores attained by products. **[D]**: product security scores by product origin. **[E]**: product security scores by antimicrobial class.

The most frequently incorporated security features were serial codes (n = 182/275, 66.2%), special random patterns (n = 35/275, 12.7%), and barcodes/QR codes (n = 27/275, 9.8%). The less commonly encountered features were watermarks (n = 8/275, 2.9%), holograms (n = 3, 1.1%) and colour shifting inks (n = 2/275, 0.7%) (**Fig 5a**). The barcodes/QR codes were mostly found on secondary package (n = 25/27, 92.6%) (**Fig 5b**) and completely absent on the tertiary packages. The presence of bar/QR codes was highest in products of foreign origin (n = 23/27, 85.2%) and exclusively on antifungals (n = 19/27, 70.4%) and antivirals (n = 8/27, 29.6%).

Additionally, most of the bar/QR codes identified (n = 21/27, 77.8%) were verifiable upon scanning (**Fig 5b**), including all antivirals (n = 8/8, 100%) and a greater portion of antifungals (n = 13/19, 68.4%). Some of the unverifiable bar/QR codes linked the products to unrelated information when searched online. Generally, product security features across the different levels of packaging was inadequate, and this observation contributed to low security scores (mean = 3.56 ± 1.59; MES = 8) (**Fig 5c**) recorded for most of the products. The security scores of the foreign products (mean = 3.93 ± 1.45) were significantly higher than those of the local products (mean = 3.04 ± 1.63, *p* < 0.001) (**Fig 5d**). Also, the scores for the antivirals (mean = 4.37 ± 1.57) was comparable to that of antifungals (mean = 3.82 ± 1.73), and significantly higher than that of antibacterials (mean = 3.51 ± 1.48, *p* = 0.049) and antiprotozoals (mean = 3.13 ± 1.51, *p* < 0.01) (**Fig 5e**).

### 3.7. Packaging quality index-based classification

**Fig 6:**
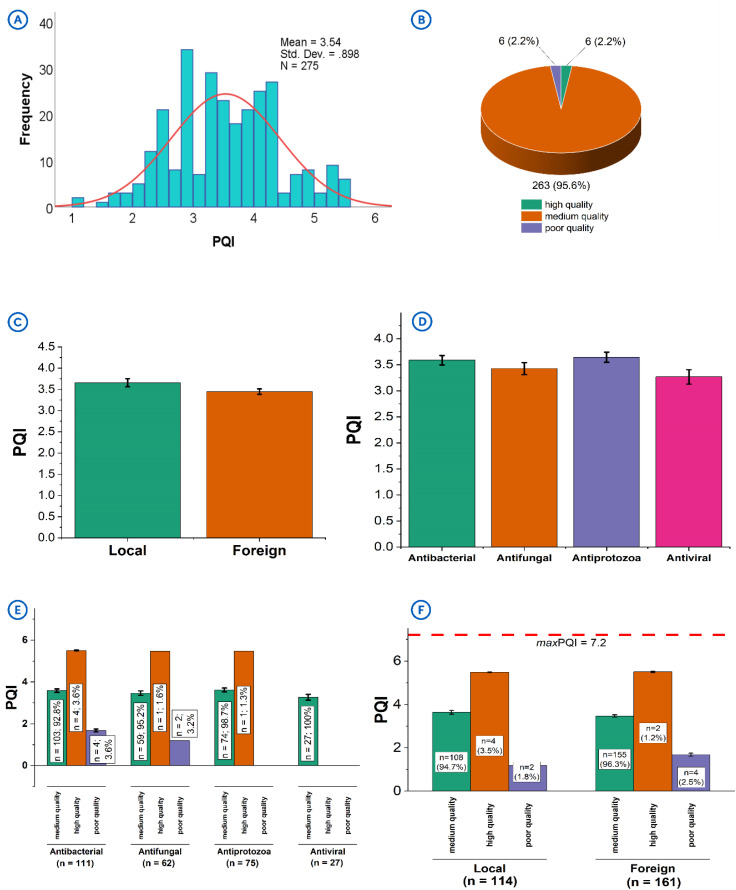
Analysis of the calculated Packaging Quality Indices (PQI) of the products. **[A]**: Distribution of PQI scores attained by products. **[B]**: Classification of quality status of the product packages. **[C]**: Comparing the PQI scores of products by their origin. **[D]**: Comparing the PQI scores by their antimicrobial class. **[E]**: Breakdown of PQI scores attained by the different antimicrobial classes. **[F]**: Breakdown of PQI scores attained by the different antimicrobials from the two origins.


The packaging quality index (PQI) of the products was calculated using Equation 2. `The PQIs calculated ranged between 1.0 and 6.0, with a mean of 3.54 ± 0.90 (**Table 1**; **Fig 6a**) and most of the packages scored medium quality PQIs (**Fig 6b**). Only a few of the product packages were considered to be of high quality (n = 6/275, 2.2%),

**Table 1:** Summary statistics of the calculated scores.

Parameter	N	Expected scores		Observed scores			
		Minimum	Maximum	Minimum	Maximum	Mean	Std. Deviation
Registration Compliance Scores	275	0	4	0	4	1.21	1.296
Language Information Quality Scores	275	0	10	3	10	9.56	0.927
Batch Information Consistency Score	275	0	10	1	10	5.83	1.720
Product Security Score	275	0	8	0	7	3.56	1.591
PQI	275	0	7.2	1	6	3.54	0.898

$$\begin{array}{cc} & Packaging\;Quality\;Index\;(PQI)=(\text{0}.\text{3}\text{5}\text{0}\times Registration\;Compliance\;Score)+(\text{0}.\text{0}\text{3}\text{0}\\ & \times Language\;\&Medical\;Information\;Quality\;Score)+(\text{0}.\text{2}\text{7}\text{1}\times Batch\;Information\;Consistency\;Score)\\ & +(\text{0}.\text{3}\text{4}\text{9}\times Product\;Security\;Score) \end{array}$$ (2)

The analysis of the PQI scores showed comparable packaging quality between products of local (3.65 ± 1.00) and foreign origin (3.45 ± 0.81; *p* = 0.058) (**Fig 6c**). Similar observation was made in the different antimicrobial classes, where antibacterials (3.59 ± 0.96), antifungals (3.43 ± 0.91), antiprotozoals (3.64 ± 0.84) and antivirals (3.27 ± 0.72) were seen to have comparable PQIs (*p* = 0.1880) (**Fig 6d**). Notable distinctions, however, were observed at the extremes of packaging quality, with products seen to possess high quality packaging (meaning, high PQIs) (n = 6) being antibacterials (n = 4/6, 66.7%) (**Fig 6e**). Only a small proportion of the antifungals (n = 1/6, 16.7) and antiprotozoals (n = 1/6, 16.7%) were assessed as high quality. Interestingly, these products were mostly of local origin (n = 4/6, 66.7%) (**Fig 6f**), and this may be due to their higher scores in registration compliance and batch information consistency. Also, notable was the fact that products with poor quality packages (n = 6) were antibacterials (n = 4/6, 66.7%) and antifungals (n = 2/6, 33.3%) (**Fig 6e**), with a high proportion of them being of foreign origin (n = 4/6, 66.7%) (**Fig 6f**). Antivirals (**Fig 6e**) failed to record any high-quality packaging, although their quality was comparable to the other antimicrobial classes.

## 4. Discussion

### 4.1. Background information about the products

The scope of antimicrobials studied directly mirrors the national picture, where antibacterials, which formed the majority of the study samples (40.4%), tend to dominate in terms of antimicrobials prescriptions and utilization both in the hospitals (outpatient and inpatient) and in the community settings [26–28]. This is probably due to their broad-spectrum effects, for example, for amoxicillin/clavulanic acid and cefuroxime, which make them suitable for several empiric treatments. Antimalarials also majority of the antiprotozoals (90.6%) probably due to their significant role in treating malaria cases in Ghana, known to be malaria-endemic [29]. For antivirals, there was a limited scope of the drugs seen in the community pharmacies. Those seen and included were mainly acyclovir (96.3%) which may be as a result of the restricted access to most antivirals and their sole availability for public health programmes in Ghana, like HIV/AIDS, hepatitis and tuberculosis.

In Ghana, English serves as the formal language for communication, and by regulation, it is expected that all medical information are provided in such medium or with appropriate translation [25]. Majority of the products complied with this regulatory requirement. For the products with non-English content (n = 6/275, 2.2%), one, failed to include an English translation (16.7%). Though in the minority, this occurrence raises concerns about the accessibility of the product’s package information to healthcare professionals and consumers which contributes to medication adherence and safety [30]. Additionally, this is at variance with the regulatory requirement for all products registered in Ghana to have their information either written in English or have English translation [25]. Given the significant market share of imported products in Ghana, and also evident in the composition of samples in this study (**Figs 1d & 1e**), it is important to intensify regulatory oversight as cases of substandard and falsified medicines in most developing countries are associated with high levels of product imports [31].

### 4.2. Registration compliance of the products

Proof of registration with the national regulatory agency, in this case, the Food and Drugs Authority (FDA) of Ghana, is a critical quality assurance requirement that must be demonstrable and evident on every product to allay the fears of medicine consumers with respect to their traceability, quality and safety [25]. While the FDA registration number is expected to be on all medicines duly registered in Ghana, it is particularly mandatory for the locally manufactured products [25]. These products are directly subjected to the local regulatory framework, hence, requiring full compliance to labeling standards, and this includes the display of the FDA registration number [25]. However, 40/114 (35.1%) of these local products did not show them (**Fig 2b**). On the other hand, imported medicines may already be registered in other jurisdictions and may sometimes benefit from regulatory flexibility with respect to local labeling requirements. This flexibility may account for the observed lower compliance rates among foreign products compared to local ones. The absence of the registration number on any product, whether of local or foreign origin, however, poses significant regulatory challenges. For the locally manufactured ones, this practice may undermine the critical regulatory framework put in place to ensure product safety, efficacy and traceability [25]. It also raises questions about the thoroughness of regulatory oversight and suggests potential lapses in enforcement mechanisms. For products of foreign origin, the absence of registration numbers complicates cross-jurisdictional verification, and this makes it difficult to establish whether such products meet both local and international quality standards [32].

Without an FDA registration number and valid registration status, health professionals and distributors are left without a vital tool to track product authenticity and approval status. This phenomenon tends to hinder efforts to detect and report potentially unregistered, substandard, or falsified medicines for further regulatory scrutiny. This practice impedes regulatory audits, which may rely on registration numbers as a primary means to monitor compliance and tracing the supply chain [4,33,34].

### 4.3. Language and information quality

Clarity, accuracy, readability and comprehensibility are crucial qualities that should characterize the information available on product packages to promote their safe use [25]. Despite the positive trends observed with language clarity, a few products had spelling and punctuation errors (2.2%), and others either had ambiguous or no appropriate medical terminologies (6.5%). Such occurrences, though few, could undermine consumer confidence and regulatory perceptions. Notwithstanding these deviations, the general observations suggested that language quality standards were highly upheld by manufacturers.

### 4.4. Batch information consistency

Batch information provides a foundation for product traceability and quality assurance, for example, in the preliminary detection of substandard and falsified medicines [35]. The observed practice of not having sufficient batch information on tertiary product packing could potentially compromise bulk product traceability during transportation and storage in medicine supply chain [35]. Batch consistency across the different packaging levels is critical for regulatory compliance, as discrepancies could raise concerns about product authenticity and quality. Inconsistent batch information, as was observed in few of the products (n = 5/275, 1.8%), compromises the ability of regulators to trace products effectively, and this may undermine efforts to identify and recall products when the need arises [36]. These inconsistencies highlight possible underlying challenges with manufacturing, packaging or labelling practices, or potential cases of product falsification, for which reason existing quality assurance systems of manufacturers should be strengthened to detect them before finished product release [37].

The significantly higher batch consistency scores for the antiprotozoals than the other antimicrobial classes (*p* < 0.01) could be attributed to the heightened regulatory focus on antiprotozoals due to their high demand, high risk of being falsified as widely reported in literature [15,38,39], and critical role they play in malaria treatment and prevention, which are public health priorities in the Sub-Saharan African region. The lower scores for the antivirals, which are mostly imported, is a cause for concern and therefore requires greater regulatory oversight to ensure consistent labelling. Addressing these gaps will enhance product traceability and support pharmacovigilance efforts across all antimicrobial classes.

### 4.5. Product security

Security features are specialized overt and covert features, design elements or digital technologies incorporated into the packaging of pharmaceutical product for the purposes of authentication and traceability along the supply chain, with the end goal of protecting them from counterfeiting or falsification [35]. Their presence enhances the integrity of products and ensures that they reach consumers in their intended quality. In the current study, security features were lacking in some product packages (10.2%, **Fig 5a**), and this could pose them at risk of falsification.

The barcodes/QR codes found mostly on the secondary package may suggest prioritizing traceability features on outer layers of products to be easily accessible to the consumer, to facilitate logistics, supply chain monitoring, and regulatory inspections [35]. The concern, however, is the complete absence of these codes on the tertiary packages encountered in the study. The presence of bar/QR codes on products of foreign origin and exclusively on antifungals and antivirals could signal the presence of stricter regulatory requirements or advanced packaging standards in the countries where the products are manufactured as compared to the local setting where there seem to be a gap in adopting traceability technologies within the local pharmaceutical industry. The exclusive association of barcodes/QR codes to antifungals and antivirals in this study could, therefore, be indicative of the high value placed on these antimicrobial classes due to their greater risk of counterfeiting or falsification [35,40].

The low product security features and scores, as seen in this study, highlight a critical component of pharmaceutical product lifecycle, which is ignored and/or poorly executed. Without verifiable security features, healthcare providers and consumers will not be able to authenticate products, thereby increasing reliance on trust rather than evidence for medicine quality assurance. This challenge can be addressed with the establishment of a track and trace system which will enable real-time verification of the authenticity of the product. While the existing system produce some sort of verification, it is not considered a robust one to assure reliable verification; the reason for which some of the products had the bar/QR codes but were not verifiable because they were not linked to any active verification platform. In this case, the codes tend to be just informational elements or internal security tools for the manufacturers instead of also serving as mechanisms to protect public health. It is, therefore, crucial for manufacturers, and regulators, especially in the local settings, to begin to prioritize product security standards that safeguard public safety. This is possible if the stakeholders work together to develop an interoperable open-access track and trace system that will not only benefit the manufacturers, but also health professionals and medicine consumers, to boost transparency and assure trust in the medicines supply-chain system.

### 4.7. Packaging quality index-based classification

Packaging plays a critical role in maintaining the integrity, authenticity, stability as well as the safety of medicines. Existing evaluations are often subjective or inconsistent. The packaging quality index (PQI) was developed to provide a standardized and objective quantitative measure to assess the quality of pharmaceutical packaging. As a composite measure, PQI integrated scores from registration compliance, language and information quality, batch information consistency, and product security. Through a systematic and iterative process involving the use of principal component analysis (PCA) to reduce data dimensionality and select the most significant independent scores, the respective weights (as a measure of their contribution to the variability in the data) were calculated as normalized sum of adjusted coefficients from PC1, PC2 and PC3 [S3–S5 Tables]. This, translated into the respective coefficients in the regression equation, which was used to calculate the PQI for each product package (Eq 2). The calculated PQI scores, therefore, allowed the classification of the quality of product packages based on established criteria and also provided for a more reliable comparison across different antimicrobial classes.

With the score ranging between 0 and 7.2 (which is the maximum PQI (maxPQI)), packages with PQI scores between 5.40–7.20 were considered to be of high quality. Packages with PQI scores between 1.80–5.40 were considered to be of moderate or intermediate quality. Packages with PQI scores less than 1.8 were considered poor quality, and such products should be investigated further either as candidates for packaging standards enhancement or as potential SF medicines. The application of this evaluation system may not only facilitate quality assurance but also inform manufacturers and stakeholders on areas for improvement in packaging standards.


$$\begin{array}{cc} & Packaging\;Quality\;Index\;(PQI)=(\text{0}.\text{3}\text{5}\text{0}\times Registration\;Compliance\;Score)\\ & +(\text{0}.\text{0}\text{3}\text{0}\times Language\;\&Medical\;Information\;Quality\;Score)\\ & +(\text{0}.\text{2}\text{7}\text{1}\times Batch\;Information\;Consistency\;Score)+(\text{0}.\text{3}\text{4}\text{9}\times Product\;Security\;Score) \end{array}$$ (3)

Applying the developed model to the products in this study resulted in the data described in **Section 3.5** of the results. Only a few of the product packages were considered to be of high quality (n = 6/275, 2.2%), suggesting that while most products met basic quality expectations, very few excelled in meeting comprehensive packaging standards. The general lack of scores approaching the maxPQI (**Table 1**; **Fig 6a**) indicates room for improvement in packaging quality, especially, for critical attributes like security features and batch information consistency.

The marginal difference between the packaging quality of the local and foreign products may reflect a high level of similarity in terms of packaging standards in respect of their origins. However, a relatively higher variation was observed among PQIs of the local products, and this could indicate some levels of inconsistencies in compliance or manufacturing practices.

The dominance of antibacterials at both ends of the packaging quality spectrum is a cause for concern which requires further investigations into the variabilities to inform targeted interventions. Antivirals (**Fig 6e**) failed to record any high-quality packaging, although their quality was comparable to the other antimicrobial classes. This may reflect some potential packaging quality gaps especially in relation to registration compliance and batch information consistency, which requires intervention given the significance of antiviral therapies in public health.

## 5. Conclusion

This study provides a comprehensive assessment of the packaging quality of antimicrobial products available within community pharmacies in the Ho municipality using packaging quality index (PQI), as an objective assessment tool. The findings indicate medium or moderate packaging quality for the majority of the products. The packaging quality of local and foreign products were comparable, with variations in some of the packaging characteristics, such as registration compliance, batch information consistency and product security. Language and batch information showed high consistency, but the lack of security measures, especially in some local products, raises concerns about brand protection and traceability of products. The PQI provides a quantitative approach to evaluate product packaging quality based on the visual and regulatory indicators. In this study, it has shown promise to support routine visual assessment. This could be considered along with laboratory analysis to screen medicinal products with quality concerns. Its application, thus, highlight the potential value of incorporating standardized assessment metrics into broader assessment strategies to gain preliminary information to warrant for further regulatory investigations. Going forward, it is important for manufacturers, especially of local origin, to incorporate security features into their packaging to increase product security and contribute to consumer safety. The observed inconsistencies in the display of registration number especially on imported products needs a critical review by regulators and manufacturers. It is important to standardize the provision of such information to enhance user confidence and help prevent unregistered or potentially unsafe versions of such medicines from entering the supply chain.

### 5.1. Limitations

This study is characterized by some limitations that need to be considered when interpreting results from the use of the developed assessment tool. While training on its use is crucial and would guarantee reproducible outcomes, it must be acknowledged that in large scale applications, some variabilities in the outcomes may be encountered. Also, the tool only assesses visual attributes of product packaging and labelling. It is, therefore, possible that due to sophistication, some falsified products with high quality packaging may escape flagging. Then again, the scope of the study did not allow for the validation of the results with pharmacopoeial tests. Thus, the use of this tool should be complementary to laboratory-based analytical techniques; it should not replace them. Also, the study took place in one municipality and so the results should be interpreted in context of the sampling performed. Another limitation observed relates to the scoring system, where due to the weights assigned to registration compliance, products with lower registration compliance (especially with the absence of registration numbers), score relatively low PQIs. Such may be the case of some foreign products which are legally imported and compliant with the country-of-origin regulations but score low PQIs because of the absence of the FDA registration number. Interpreting such cases therefore requires contextual judgement, preferably in consultation with the national regulator to avoid misclassification. Notwithstanding all these limitations, the findings provide valuable insights into the packaging and labeling quality and justify the benefit that the developed tool has in serving as an early warning tool to detect suspicious antimicrobial and other medicinal products.

## Supporting information

S1 Fig Biplot from the principal component analysis involving the four categories of scores to investigate the relationship among them in respect of the packaging quality assessment. (DOCX)

S1 Table Assessment Form for Packages and Labels of Antimicrobial Drugs in Ho Municipality. (DOCX)

S2 Table Scoring System for Visual Assessment of Product Packages. (DOCX)

S3 Table Report from the PCA involving the four categories of scores in the packaging quality assessment. (DOCX)

S4 Table Extracted Eigenvectors for the principal components in the PCA. (DOCX)

S5 Table Variance contribution from the principal components in the PCA. (DOCX)
