# Supplementary material for: Visual assessment of antimicrobial medicine packaging and labeling quality in pharmacies of Ho Municipality, Ghana
Source: PLoS One. 2026 Feb 13;21(2):e0342484. doi: 10.1371/journal.pone.0342484 (PMC12904372; doi:10.1371/journal.pone.0342484)
Supplement: S1 Fig — (DOCX) [file pone.0342484.s001.docx]

***Supplementary Information***

**Visual Assessment of Antimicrobial Medicine Packaging and Labeling Quality in Pharmacies of Ho Municipality, Ghana**

Emmanuel Orman^1*^, Bridget Dzidzinu Ankah^1^, David Oteng^1^, David Mccarthur^2^, Thelma Alalbila Aku^1^, Araba Ata Hutton-Nyameaye^1^, Jonathan Jato^1^, Hayford Odoi^1^, Samuel Owusu Somuah^1^, Issaka Nii Amu Collison-Cofie^3^, Yogini H Jani^4,5^, Cornelius Dodoo^1^

*^1^School of Pharmacy, University of Health and Allied Sciences, Ho, Ghana*

*^2^Pharmacy Department, Ho Teaching Hospital, Ho, Ghana*

*^3^Food and Drugs Authority, Ho, Ghana*

*^4^ School of Pharmacy, University of London, London, UK*

*^5^Centre for Medicines Optimisation Research and Education, UCLH NHS Foundation Trust, London, UK*

**Correspondence**

Department of Pharmaceutical Chemistry, School of Pharmacy, University of Health and Allied Sciences, PMB 31, Ho, Ghana. [eorman@uhas.edu.gh](mailto:eorman@uhas.edu.gh)

**Development of the Packaging Quality Index**

1. ***Exploratory analysis of the scores from the visual assessment using PCA***


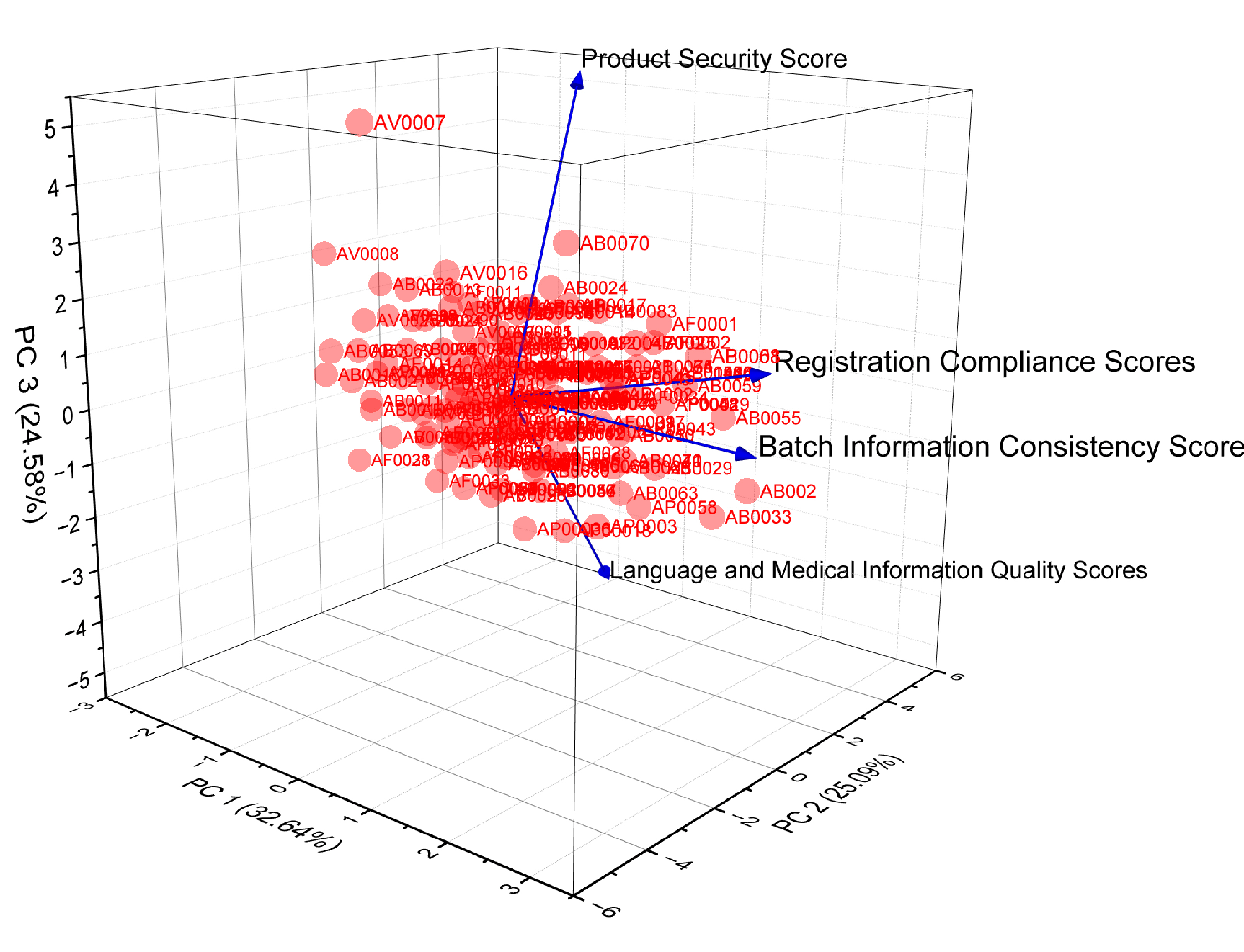


**S1 Figure**: Biplot from the principal component analysis involving the four categories of scores to investigate the relationship among them in respect of the packaging quality assessment.
