# Supplementary material for: Visual assessment of antimicrobial medicine packaging and labeling quality in pharmacies of Ho Municipality, Ghana
Source: PLoS One. 2026 Feb 13;21(2):e0342484. doi: 10.1371/journal.pone.0342484 (PMC12904372; doi:10.1371/journal.pone.0342484)
Supplement: S1 Table — (DOCX) [file pone.0342484.s002.docx]

***Supplementary Information***

**Visual Assessment of Antimicrobial Medicine Packaging and Labeling Quality in Pharmacies of Ho Municipality, Ghana**

Emmanuel Orman^1*^, Bridget Dzidzinu Ankah^1^, David Oteng^1^, David Mccarthur^2^, Thelma Alalbila Aku^1^, Araba Ata Hutton-Nyameaye^1^, Jonathan Jato^1^, Hayford Odoi^1^, Samuel Owusu Somuah^1^, Issaka Nii Amu Collison-Cofie^3^, Yogini H Jani^4,5^, Cornelius Dodoo^1^

*^1^School of Pharmacy, University of Health and Allied Sciences, Ho, Ghana*

*^2^Pharmacy Department, Ho Teaching Hospital, Ho, Ghana*

*^3^Food and Drugs Authority, Ho, Ghana*

*^4^ School of Pharmacy, University of London, London, UK*

*^5^Centre for Medicines Optimisation Research and Education, UCLH NHS Foundation Trust, London, UK*

**Correspondence**

Department of Pharmaceutical Chemistry, School of Pharmacy, University of Health and Allied Sciences, PMB 31, Ho, Ghana. [eorman@uhas.edu.gh](mailto:eorman@uhas.edu.gh)

**S2 Table:** Scoring System for Visual Assessment of Product Packages

1. Registration Compliance Scores

| **Sr No** | **Question** | **Response** | **Score** | **Cumulative score** |
| --- | --- | --- | --- | --- |
| 1 | Is the language used English | Yes | 1 | 1 |
|  |  | No | 0 |  |
| 2 | If non-English, is an English translation included in the package or label? | Yes | 1 |  |
|  |  | No | 0 |  |
|  |  | NA | 0 |  |
| 3 | Is the FDA number present on the package? | Yes | 1 | 2 |
|  |  | No | 0 |  |
| 4 | Is the registration status valid as at the time of study? (confirm with FDA list of Registered products online) | Yes | 1 | 3 |
|  |  | No | 0 |  |
| 5 | Is the FDA Number present on the different levels of the package? | Yes | 1 | 4 |
|  |  | No | 0 |  |
|  |  | NA | 0 |  |
| 6 | Is the FDA number on the different levels of package the same? | Yes | 1 | 5 |
|  |  | No | 0 |  |
|  |  | NA | 0 |  |
|  | ***Registration Compliance Score*** |  |  | **5** |

1. Language and Medical Information Scores

| **Sr No** | **Question** | **Response** | **Score** | **Cumulative score** |
| --- | --- | --- | --- | --- |
| 1 | Language is clear and grammatically correct. | Excellent | 4 | 4 |
|  |  | Good | 3 |  |
|  |  | Fair | 2 |  |
|  |  | Poor | 1 |  |
| 2 | Language is comprehensible and suitable for the target audience | Excellent | 4 | 8 |
|  |  | Good | 3 |  |
|  |  | Fair | 2 |  |
|  |  | Poor | 1 |  |
| 3 | Does the information use appropriate medical terminology and avoid ambiguity? | Yes | 1 | 9 |
|  |  | No | 0 |  |
| 4 | Are there any spelling or punctuation errors in the English text? | Yes | 0 | 10 |
|  |  | No | 1 |  |
|  | ***Language and Medical Information Score*** |  |  | **10** |

1. Batch Information Consistency Scores

| **Sr No** | **Question** | **Response** | **Score** | **Cumulative score** |
| --- | --- | --- | --- | --- |
| 1 | Batch number present on primary package | Yes | 1 | 1 |
|  |  | No | 0 |  |
|  |  | NA | 0 |  |
| 2 | Manufacturing date present on primary package | Yes | 1 | 2 |
|  |  | No | 0 |  |
|  |  | NA | 0 |  |
| 3 | Expiry date present on primary package | Yes | 1 | 3 |
|  |  | No | 0 |  |
|  |  | NA | 0 |  |
| 4 | Batch number present on secondary package | Yes | 1 | 4 |
|  |  | No | 0 |  |
|  |  | NA | 0 |  |
| 5 | Manufacturing date present on secondary package | Yes | 1 | 5 |
|  |  | No | 0 |  |
|  |  | NA | 0 |  |
| 6 | Expiry date present on secondary package | Yes | 1 | 6 |
|  |  | No | 0 |  |
|  |  | NA | 0 |  |
| 7 | Batch number present on tertiary package | Yes | 1 | 7 |
|  |  | No | 0 |  |
|  |  | NA | 0 |  |
| 8 | Manufacturing date present on tertiary package | Yes | 1 | 8 |
|  |  | No | 0 |  |
|  |  | NA | 0 |  |
| 9 | Expiry date present on tertiary package | Yes | 1 | 9 |
|  |  | No | 0 |  |
|  |  | NA | 0 |  |
| 10 | Batch information on the different levels of package are the same | Yes | 1 | 10 |
|  |  | No | 0 |  |
|  |  | NA | 0 |  |
|  | ***Batch Information Consistency Score*** |  |  | **10** |

1. Product Security Scores

| **Sr No** | **Question** | **Response** | **Score** | **Cumulative score** |
| --- | --- | --- | --- | --- |
| 1 | Number of security features | No security feature | 0 | 3 |
|  |  | 1 to 2 security features | 1 |  |
|  |  | 3 to 4 security features | 2 |  |
|  |  | 5 or more security features | 3 |  |
| 2 | Verifiable barcode/QR code on scanning | Yes | 1 | 4 |
|  |  | No | 0 |  |
|  |  | NA | 0 |  |
| 3 | Presence of one or more temper-resistant features | No feature | 0 | 5 |
|  |  | 1 or more feature | 1 |  |
| 4 | Complexity of serialization | No serial number | 0 | 8 |
|  |  | NA | 0 |  |
|  |  | Numbers alone | 1 |  |
|  |  | Alphabets + numbers | 2 |  |
|  |  | Alphabets + numbers + special characters | 3 |  |
|  | ***Product Security Score*** |  |  | **8** |
