# Supplementary material for: Visual assessment of antimicrobial medicine packaging and labeling quality in pharmacies of Ho Municipality, Ghana
Source: PLoS One. 2026 Feb 13;21(2):e0342484. doi: 10.1371/journal.pone.0342484 (PMC12904372; doi:10.1371/journal.pone.0342484)
Supplement: S3 Table — (DOCX) [file pone.0342484.s004.docx]

***Supplementary Information***

**Visual Assessment of Antimicrobial Medicine Packaging and Labeling Quality in Pharmacies of Ho Municipality, Ghana**

Emmanuel Orman^1*^, Bridget Dzidzinu Ankah^1^, David Oteng^1^, David Mccarthur^2^, Thelma Alalbila Aku^1^, Araba Ata Hutton-Nyameaye^1^, Jonathan Jato^1^, Hayford Odoi^1^, Samuel Owusu Somuah^1^, Issaka Nii Amu Collison-Cofie^3^, Yogini H Jani^4,5^, Cornelius Dodoo^1^

*^1^School of Pharmacy, University of Health and Allied Sciences, Ho, Ghana*

*^2^Pharmacy Department, Ho Teaching Hospital, Ho, Ghana*

*^3^Food and Drugs Authority, Ho, Ghana*

*^4^ School of Pharmacy, University of London, London, UK*

*^5^Centre for Medicines Optimisation Research and Education, UCLH NHS Foundation Trust, London, UK*

**Correspondence**

Department of Pharmaceutical Chemistry, School of Pharmacy, University of Health and Allied Sciences, PMB 31, Ho, Ghana. [eorman@uhas.edu.gh](mailto:eorman@uhas.edu.gh)

**S4 Table:** Extracted Eigenvectors for the principal components in the PCA

|  | **Coefficients of PC1** | **Coefficients of PC2** | **Coefficients of PC3** |
| --- | --- | --- | --- |
| Registration Compliance Scores | 0.6756 | 0.22105 | 0.13293 |
| Language and Medical Information Quality Scores | -0.1416 | 0.72774 | -0.67019 |
| Batch Information Consistency Score | 0.6928 | 0.12508 | -0.04712 |
| Product Security Score | -0.20869 | 0.63709 | 0.72867 |
