# Supplementary material for: Visual assessment of antimicrobial medicine packaging and labeling quality in pharmacies of Ho Municipality, Ghana
Source: PLoS One. 2026 Feb 13;21(2):e0342484. doi: 10.1371/journal.pone.0342484 (PMC12904372; doi:10.1371/journal.pone.0342484)
Supplement: S4 Table — (DOCX) [file pone.0342484.s005.docx]

***Supplementary Information***

**Visual Assessment of Antimicrobial Medicine Packaging and Labeling Quality in Pharmacies of Ho Municipality, Ghana**

Emmanuel Orman^1*^, Bridget Dzidzinu Ankah^1^, David Oteng^1^, David Mccarthur^2^, Thelma Alalbila Aku^1^, Araba Ata Hutton-Nyameaye^1^, Jonathan Jato^1^, Hayford Odoi^1^, Samuel Owusu Somuah^1^, Issaka Nii Amu Collison-Cofie^3^, Yogini H Jani^4,5^, Cornelius Dodoo^1^

*^1^School of Pharmacy, University of Health and Allied Sciences, Ho, Ghana*

*^2^Pharmacy Department, Ho Teaching Hospital, Ho, Ghana*

*^3^Food and Drugs Authority, Ho, Ghana*

*^4^ School of Pharmacy, University of London, London, UK*

*^5^Centre for Medicines Optimisation Research and Education, UCLH NHS Foundation Trust, London, UK*

**Correspondence**

Department of Pharmaceutical Chemistry, School of Pharmacy, University of Health and Allied Sciences, PMB 31, Ho, Ghana. [eorman@uhas.edu.gh](mailto:eorman@uhas.edu.gh)

**S5 Table:** Variance contribution from the principal components in the PCA

| **PC** | **% Variance Explained** | **Square Root of Variance** |
| --- | --- | --- |
| PC 1 | 32.64% | $\sqrt{0.3264}=0.5715$ |
| PC 2 | 25.09% | $\sqrt{0.2509}=0.5010$ |
| PC 3 | 24.58% | $\sqrt{0.2458}=0.4958$ |

Calculating the adjusted coefficients

| **Variable** | **PC1 Coeff × 0.5715** | **PC2 Coeff × 0.501** | **PC3 Coeff × 0.4958** |
| --- | --- | --- | --- |
| FDA Registration and Compliance | 0.6756 × 0.5715 = 0.3863 | 0.22105 × 0.501 = 0.1107 | 0.13293 × 0.4958 = 0.0659 |
| Language and Information Quality | -0.1416 × 0.5715 = -0.0810 | 0.72774 × 0.501 = 0.3647 | -0.67019 × 0.4958 = -0.3325 |
| Batch Information & Consistency | 0.6928 × 0.5715 = 0.3960 | 0.12508 × 0.501 = 0.0627 | -0.04712 × 0.4958 = -0.0234 |
| Security Feature Score | -0.20869 × 0.5715 = -0.1192 | 0.63709 × 0.501 = 0.3194 | 0.72867 × 0.4958 = 0.3614 |

**Step 2**

The adjusted coefficients across PC1, PC2 and PC3 were summed together and converted to absolute values.

| **Variable** | **Sum of Adjusted Coefficients** | **\| Absolute Sum \|** |
| --- | --- | --- |
| FDA Registration and Compliance | 0.3863 + 0.1107 + 0.0659 = 0.5629 | 0.5629 |
| Language and Information Quality | -0.0810 + 0.3647 - 0.3325 = -0.0488 | 0.0488 |
| Batch Information & Consistency | 0.3960 + 0.0627 - 0.0234 = 0.4353 | 0.4353 |
| Security Feature Score | -0.1192 + 0.3194 + 0.3614 = 0.5616 | 0.5616 |

**Step 3**

The absolute coefficients were then normalized to obtain the final weights using the formula:

$${Weight}_{x}=\frac{\left| {Sum}_{x} \right|}{\sum\left| {Sum}_{x} \right|}$$

Total Sum = 0.5629 + 0.0488 + 0.4353 + 0.5616 = 1.6086

| **Variable** | **Final Weight** |
| --- | --- |
| FDA Registration and Compliance | 0.5629/1.6086 = 0.350 |
| Language and Information Quality | 0.0488/1.6086 = 0.030 |
| Batch Information & Consistency | 0.4353/1.6086 = 0.271 |
| Security Feature Score | 0.5616/1.6086 = 0.349 |

Final equation to calculate PQI of the product packages

$$Packaging Quality Index (PQI)=\left( 0.350\times Registration Compliance Score \right)+\left( 0.030\times Language \& Information Quality Score \right)+\left( 0.271\times Batch Consistency Score \right)+(0.349\times Packaging Security Score)$$
